# Neurological Disease in Lupus: Toward a Personalized Medicine Approach

**DOI:** 10.3389/fimmu.2018.01146

**Published:** 2018-06-06

**Authors:** Sarah McGlasson, Stewart Wiseman, Joanna Wardlaw, Neeraj Dhaun, David P. J. Hunt

**Affiliations:** ^1^MRC Institute of Genetics and Molecular Medicine, Edinburgh, United Kingdom; ^2^The UK Dementia Research Institute, University of Edinburgh, Edinburgh, United Kingdom; ^3^The Anne Rowling Clinic, University of Edinburgh, Edinburgh, United Kingdom; ^4^Centre for Cardiovascular Science, Queen’s Medical Research Institute, University of Edinburgh, Edinburgh, United Kingdom

**Keywords:** neurolupus, personalized medicine, lupus erythematosus, systemic, targeted therapy, interferon type I

## Abstract

The brain and nervous system are important targets for immune-mediated damage in systemic lupus erythematosus (SLE), resulting in a complex spectrum of neurological syndromes. Defining nervous system disease in lupus poses significant challenges. Among the difficulties to be addressed are a diversity of clinical manifestations and a lack of understanding of their mechanistic basis. However, despite these challenges, progress has been made in the identification of pathways which contribute to neurological disease in SLE. Understanding the molecular pathogenesis of neurological disease in lupus will inform both classification and approaches to clinical trials.

## Introduction

Systemic lupus erythematosus (SLE, lupus) is a multiorgan autoimmune disease, initially described on the basis of its cutaneous manifestations (1). During the nineteenth century, the true multisystem nature of the disease was recognized with the initial descriptions of severe brain involvement (2, 3). The first dedicated clinical studies of neurological dysfunction in lupus were reported in 1945 by David Daly (4). His observations were astute, noting a high degree of heterogeneity in the neurological manifestations, and a prominent contribution of neurovascular disease. Over the following decades, the effects of lupus on all levels of the nervous system have been recognized.

**Figure 1:**
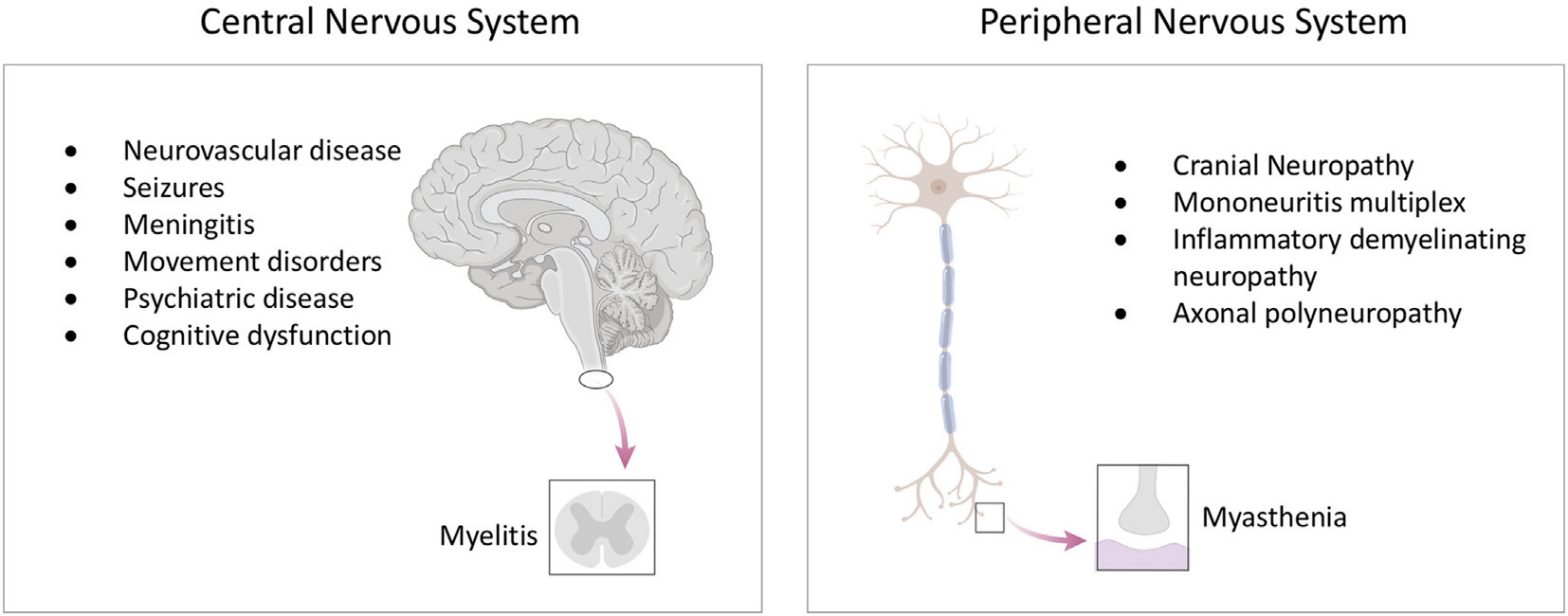
The spectrum of neurological disease in lupus. Lupus can affect all levels of the nervous system, including the brain and spinal cord, as well as the peripheral nervous system. See text for detailed descriptions of individual syndromes.

The diversity of neurological disease in lupus stimulated calls for a classification system to facilitate its clinical and scientific study (5). In 1999, the American College of Rheumatology (ACR) developed criteria for case definitions for neurolupus (6). These broadly distinguish between complications which affect the central nervous system and peripheral nervous system (Table 1 and Figure 1). While minor modifications have been proposed to these criteria, they have remained largely unchanged for almost two decades (7, 8). Neurological events have also been incorporated into diagnostic criteria for lupus, as well as outcome metrics such as the SLICC/ACR Damage index (9, 10).

**Table 1:** Clinical syndromes seen in people with systemic lupus erythematosus.

	Syndrome	Implicated mechanisms and potential therapeutic targets
CNS	Large and small vessel disease	Large vessel atheromatous disease (57) Accelerated cerebral small vessel disease (18) Antiphospholipid antibodies (49)
	Seizures	Unknown (69)
	Myelopathy	Antibody-mediated [aquaporin-4, myelin oligodendrocyte glycoprotein (MOG)] (21, 147, 148) Vascular
	Meningitis	Unknown (78)
	Movement disorder	Unknown (84)
	Demyelinating syndrome	Not clearly associated with SLE (89)
	Headache	Not clearly associated with SLE (90)
	Psychiatric disease	Cytokine dysregulation (107) Antibody-mediated (NMDA-R, Ribosomal-P) (97)
	Cognitive dysfunction	Cytokine dysregulation (38) Small vessel disease (18, 61)
PNS	Peripheral neuropathy	Vasculitis (124) Antibody-mediated (ganglioside) (149)
	Cranial neuropathy	Vasculitis Antibody-mediated (aquaporin-4/MOG) (132, 150)
	Myasthenia Gravis	Antibody-mediated (anti-AChR) (151)

The development of the ACR neurolupus definitions helped stimulate the epidemiological study of neurological disease in lupus, and has demonstrated that nervous system involvement is a major negative determinant of quality of life (11–13). However, such studies have highlighted one of the major problems in the field—the issue of establishing a causal association between a neurological syndrome and lupus (14). For example, the ACR criteria include terms such as *headache* and *mood disorder* which are highly prevalent in the general population and observed at similar frequency in healthy, matched controls, as well as patients with other chronic inflammatory diseases (15). As such they are less likely to be caused directly by lupus. When “minor events” such as headache and anxiety disorders are included in population studies, then 40% of patients had at least one neuropsychiatric event (12). Exclusion of minor symptomatology leads to much improved specificity of the criteria (15). Neurological manifestations can occur at any stage of disease. Longitudinal studies of newly diagnosed patients show that neurological events attributable to lupus can occur around the time of diagnosis in approximately 5–10% of cases (16). Prospective studies show that major neurological events develop in about 5% of patients with SLE, followed over 3 years (17). Magnetic resonance imaging evidence (MRI) of brain changes indicating microvascular disease can develop early in disease course and in young patients (18, 19).

**Figure 2:**
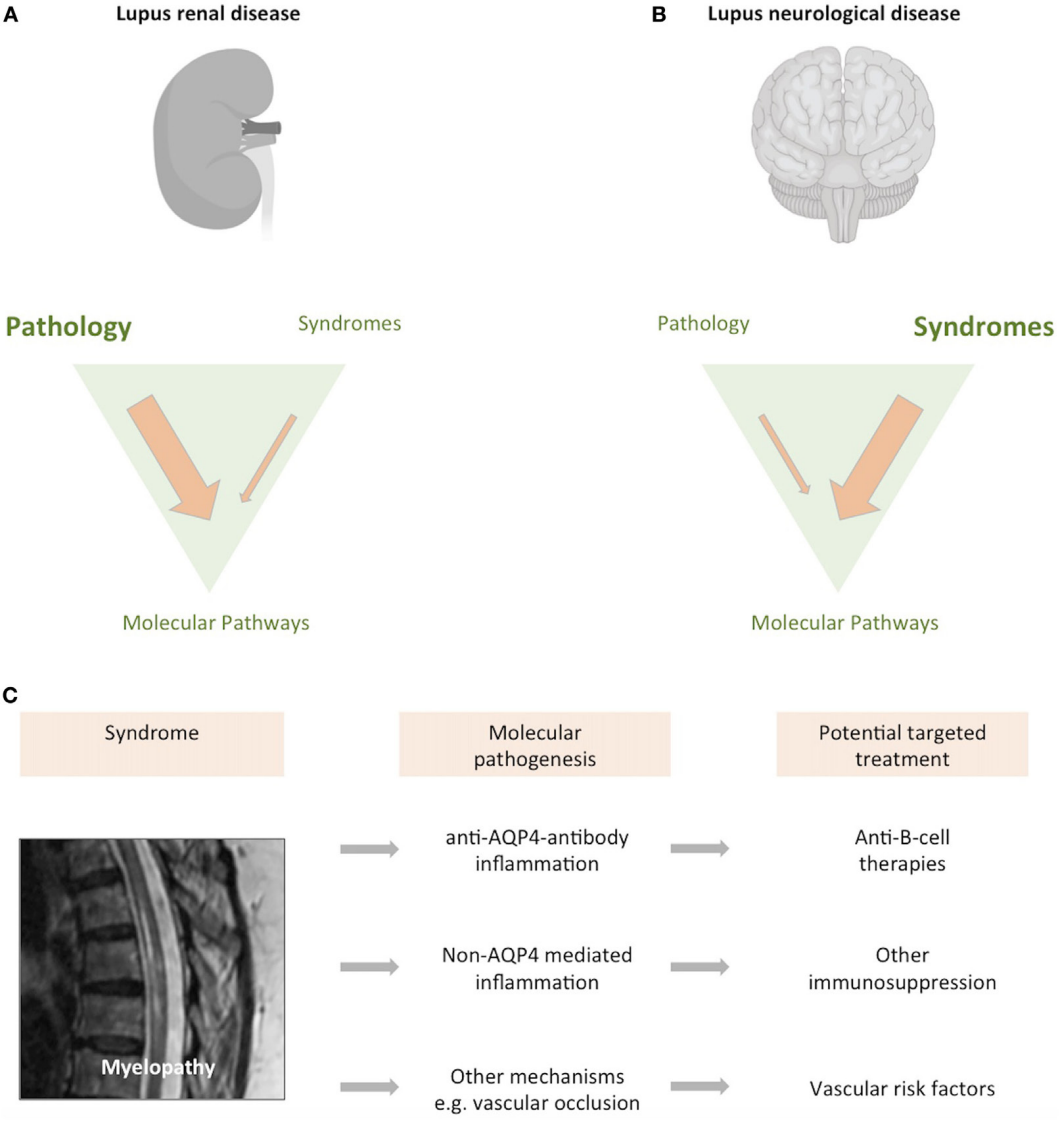
Classifying neurological disease in lupus. Both the brain and kidney can be severely affected in up to 10% of patients with lupus. **(A)** While lupus nephritis can present with different clinical syndromes, it is largely defined by a pathological classification of the renal biopsy. Image created with Biorender. **(B)** In contrast, neurological forms of lupus are usually classified according to neurological syndrome, and pathological material is rarely obtained. **(C)** We have an increasingly precise understanding of the syndrome previously described as “lupus myelopathy,” T2-weighted magnetic resonance imaging of long inflammatory lesion in person with lupus shown, with high signal from within the thoracic spinal cord. A proportion of such spinal cord presentations are driven by antibodies directed against aquaporin-4, a glial water channel (21). Other cases are caused by spinal cord inflammation without these antibodies, while some cases are associated with spinal cord ischemia (22, 23). Each of these causes may require consideration of differing therapeutic approaches. As such, what was previously considered a single disease entity can be caused by differing pathogenic mechanisms, with implications for treatment and clinical trial design.

Much of the difficulty in classification stems from a comparative lack of understanding as to how neurological disease develops in people with lupus. It is notable that the ACR definitions focus largely on neurological syndromes, rather than pathophysiological mechanisms. This is in major contrast to renal lupus, where pathophysiological classification influences treatment and prognosis (Figure 2) (20). With the development of increasingly targeted treatments, an understanding of the molecular pathogenesis of brain disease is ever more important if it is to inform clinical trial design and, ultimately an individualized therapeutic approach.

## Pathophysiology of Neurological Disease in Lupus

### Genetics

Genome-wide association studies of large cohorts of lupus patients have identified an increasing number of associations with pathways involved in both the innate and adaptive immune systems (24). However, to date there has been little dedicated genetic study of neurolupus. An evaluation of *TREX1*, a 3′–5′ exonuclease associated with SLE (25, 26), revealed a common risk haplotype in lupus patients with brain manifestations, particularly seizures (27, 28). While these mechanistic insights are of interest, testing of *TREX1* is unlikely to be of clinical utility (27, 29) given the relatively high frequency of variants in the general population (30).

### Cytokines

There is dysregulation of multiple cytokine pathways in patients with SLE (31), and recent work has focused on the extent to which these pathways might contribute to brain damage. Approximately 80% of individuals with lupus have aberrant activation of their type I interferon pathway, identified by either a transcriptomic signature, or ultrasensitive detection of the interferon-alpha proteins (32, 33). Detailed longitudinal studies have shown that activation of this pathway influences lupus disease phenotype (33).

The ability of type I interferon proteins to cause brain damage and affect mood is well documented in clinical trials of recombinant type I interferon proteins (34–36). Activation of the type I interferon response in the post-mortem brains of lupus patients has been shown (37), and multiple cell types within the brain, including endothelial cells, microglia, and neurons, respond to type I interferon activation.

Many other cytokines are dysregulated in SLE, with potential neurotoxic effects. For example, IL-6 has been associated with cognitive dysfunction in these patients and causes brain disease in brain-targeted overexpression experiments (38, 39). Type II interferons, interleukins (IL-2, IL-12, IL-18, IL-23), and TNF cytokine families are all dysregulated in lupus and their roles in brain disease are being evaluated (40).

### Inflammatory Cells

Although B cells and T cells undoubtedly play an important role in the pathogenesis of SLE, neuropathological analyses in individuals with lupus show little in the way of immune cell infiltration within the brain (41). This contrasts with other neuroinflammatory diseases such as multiple sclerosis (MS) where abundant B and T cells are found within inflammatory brain lesions (42). There has been an increasing focus on how brain-resident immune cells, such as microglia, might mediate brain disease. Recent elegant studies have shown that microglia are sensitive to elevated circulating cytokines such as type I interferon, and the resulting activation can lead to activation of a number of effector pathways within these cells, including the ability to engulf and “prune” synaptic connections (37, 43). These studies show how dysregulated cytokines can cause structural brain damage by manipulating the normal physiological processes of brain-resident immune cells.

### Antibodies

Antibodies are a major mediator of organ damage in SLE, and antibodies directed against multiple brain antigens are frequently produced (44). The extent to which such antibodies cause neurological disease remains to be fully determined. In some cases, for example, antibodies directed against the astrocytic water channel aquaporin-4 (AQP4), there is evidence to support a causal relationship with spinal cord and optic nerve inflammation (21, 45). Antibodies against neuronal cell surface proteins such as the NMDA-receptor (NMDA-R) have also been described in lupus, but a causal association with neurological symptomatology is less clear, despite their ability to mediate brain disease in animal models. Although anti-NMDA-R antibodies can cause a very distinct clinical phenotype of autoimmune encephalitis (46), this syndrome is rarely seen in SLE, and the degree to which lower titers of such antibodies can cause neuropsychiatric dysfunction outside this clinical picture is unclear (47). Interestingly, more classic lupus-associated antibodies directed against nucleic acids, can also cross-react with NMDA-R epitopes and cause neurological dysfunction in rodent models (48). In patients with SLE who have co-existing antiphospholipid syndrome there is a role for antiphospholipid antibodies in the mediation of thrombotic events including intracranial thromboembolism (49). Therefore, a broad spectrum of antibodies is implicated in the pathogenesis of neurolupus, though neurological expertise may be needed in their interpretation.

### Pathology and Imaging

**Figure 3:**
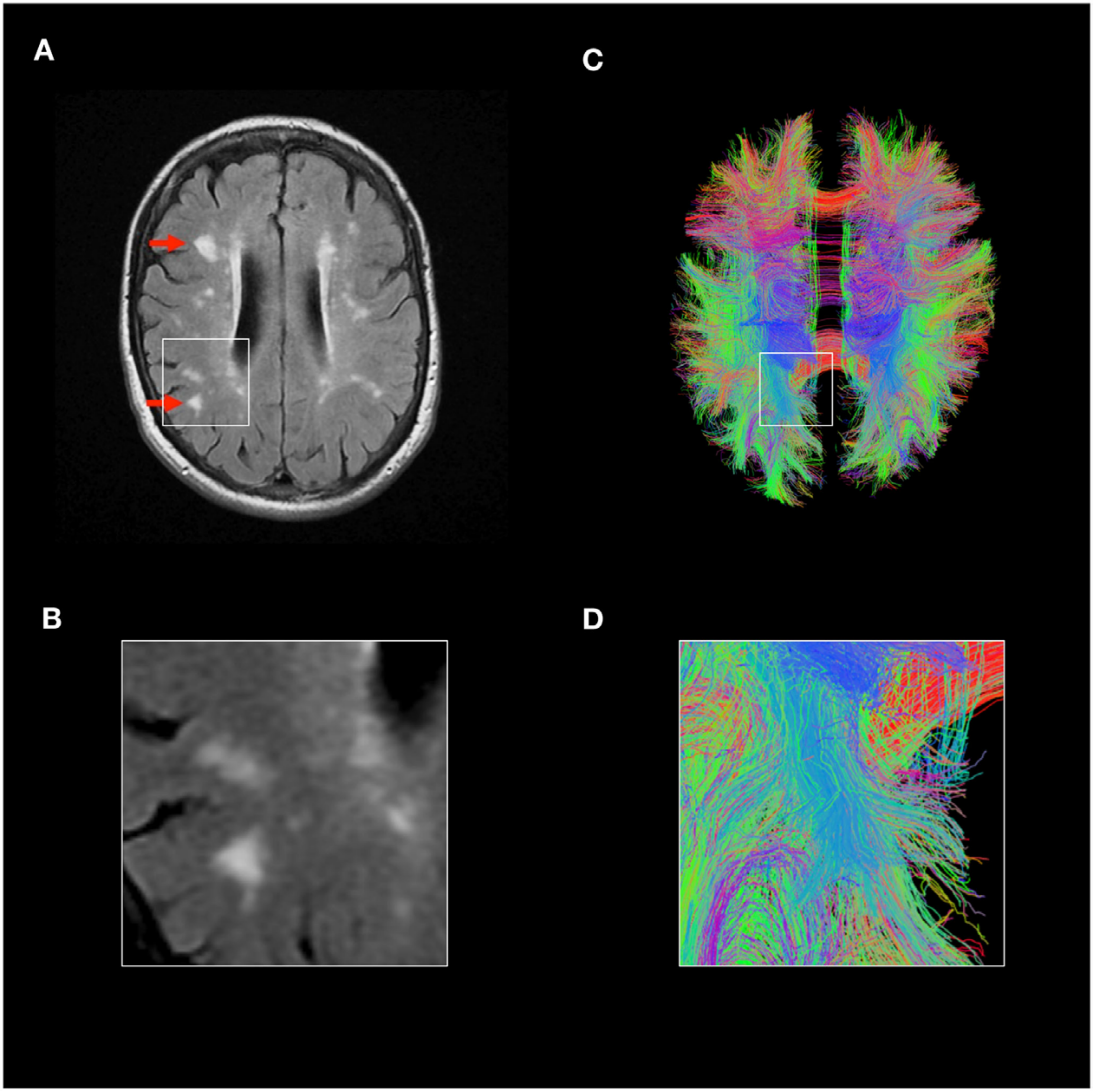
Magnetic resonance imaging (MRI) imaging in lupus brain disease. **(A,B)** Fluid-attenuated inversion recovery MRI scan of a representative individual with lupus, showing accelerated cerebral small vessel disease, highlighted red arrows. **(C,D)** Advanced MRI techniques such as diffusion tensor imaging and tractography can allow identification of individual white matter tracts and parameters such as mean diffusivity can identify microstructural disease. Tractography images of lupus patient shown, each line represents individual white matter tract. Credit: Mark Bastin, Joanna Wardlaw, and Stewart Wiseman.

Brain biopsies are performed rarely in people with lupus. Consequently, much of our understanding of the pathological basis of neurolupus comes from post-mortem studies, which introduce a bias toward severe disease. The first dedicated studies identified prominent cerebral small vessel disease as a major neuropathological feature in most cases (50). Importantly, this is not a small vessel vasculitis, but rather a noninflammatory microangiopathy associated with microinfarction (50). Pathological changes of small blood vessels include necrosis of the vessel wall, endothelial cell proliferation, and hypertrophy (41, 50, 51). Subsequent studies have confirmed these findings (52, 53). Paired pathology-imaging studies show that these cerebral small vessel lesions seen on brain pathology correspond to “white matter hyperintensities” identifiable on MRI of the brain (54). These MRI abnormalities are seen in the majority of people with lupus, even with mild neurological symptomatology (Figure 3A) (18). Sophisticated MRI imaging techniques such as diffusion imaging and quantitative tractography can map the brain’s white matter tracts and have identified evidence of microstructural damage in SLE (Figure 3C), although robust association between such changes and neurological dysfunction remains unclear (38).

## Clinical Approach in Neurolupus

The European League against Rheumatism recommendations for management of neurolupus emphasizes the importance of careful evaluation of new neurological events in individuals with SLE (55). It is important to remember that neurological symptoms may not be caused by lupus, and may simply represent highly prevalent neurological disease such as migraine or tension headache. Furthermore neurological symptoms may be caused directly or indirectly by drug therapies (14, 56). As such investigation of these symptoms should involve a detailed history, careful examination and further investigation where indicated, including MRI scan, cerebrospinal fluid analysis, and neurophysiology (56). Multidisciplinary discussion with a neurologist with an interest in neuroinflammatory disease and SLE can help.

### Recognized Clinical Syndromes

The recognized clinical neurological syndromes associated with lupus are based loosely on the framework of the ACR criteria.

#### Stroke

The earliest descriptions of lupus brain disease emphasized a prominent role for neurovascular disease (4). Subsequent studies have shown that stroke occurs more frequently in people with SLE than in the general population, with ischemic stroke developing in up to 20% of lupus patients (57–61). This observation of an increased stroke risk has been confirmed in large prospective registry based studies (59) and meta-analyses (61). Recognized risk factors, such as hypertension, smoking, and hypercholesterolemia may play an important role in this increased risk (60), but do not fully account for the excess of cases, implicating an additional inflammatory etiology (62). As such, addressing the modifiable stroke risk factors of smoking, diet, and blood pressure, is an important priority for lupus patients. Patients with lupus who present with stroke should be carefully evaluated for the antiphospholipid syndrome, given that this may direct a different strategy based on anti-coagulation rather than anti-platelet therapies. Intracranial vasculitis causing stroke—either ischemic or subarachnoid hemorrhage—is rare in SLE, but can sometimes occur and may be identified by abnormal angiographic appearances or biopsy (63–65), highlighting the heterogeneity of underlying mechanisms which drive neurovascular disease in lupus.

#### Small Vessel Disease (SVD)

Cerebral SVD is a disorder of the brain’s perforating arterioles with typical MRI brain imaging features which include white matter hyperintensities (WMH, Figure 3A). Such appearances can occasionally cause diagnostic confusion with MS, although improved imaging should aid the distinction. Accelerated cerebral SVD is a major cause of dementia in the general population, although the neurological significance of these findings in lupus remains to be determined (18). Quantified MRI brain studies of individuals with lupus show significantly accelerated cerebral SVD, suggesting that this is the most frequently observed radiological–pathological brain abnormality in lupus (41, 54, 66), seen even in patients with mild and inactive disease (18). It is likely that inflammatory mediators such as cytokines play a direct role (67), though the precise factors—and whether they might be more accurately targeted—remain to be determined.

#### Seizures

Seizures can occur in approximately 5% of individuals with lupus. These are often generalized, though can also be of focal onset (68, 69). It remains unclear as to whether such events represent a form of autoimmune epilepsy, or a lowered seizure threshold. Seizures can also occur in the context of underlying disorders, such as infection, macrophage activation syndrome (MAS) (70), or posterior reversible encephalopathy syndrome (PRES) (71), highlighting the need for appropriate investigation of seizures depending on the clinical context. There is no clear association between seizures and autoantibody formation, including the potentially epileptogenic anti-NMDA-R antibody (68). While recurrence rate of seizures appears to relatively low (69), large-scale epidemiological analyses of large databases confirm higher rates of epilepsy in people with lupus (72). Seizures should be carefully evaluated with a neurologist for underlying cause and use of anticonvulsant agents discussed in those at high risk of seizure recurrence. If anticonvulsant medication is used, particular attention may need to be paid to issues such as drug interactions and teratogenicity.

#### Myelopathy

Spinal cord disease is an uncommon but serious neurological complication in people with lupus. Over the past decade, the identification of pathogenic antibodies against glial antigens such as the AQP4 water channel has demonstrated that “lupus myelitis” can, in part, be explained by concomitant neuromyelitis optica spectrum disorder (NMOSD) (73). These autoantibodies, together with anti-myelin oligodendrocyte glycoprotein (MOG) antibodies, should be tested in spinal cord presentations, especially in the context of “longitudinally extensive transverse myelitis” where inflammation extends over at least three vertebral segments (74). The presence of AQP4 antibodies is associated with a risk of relapse and immunosuppression is typically used to prevent further events. The B-cell depleting monoclonal antibody rituximab is increasingly used as a first- or second-line agent (21, 74, 75). Antibodies against AQP4 can be generated in people with lupus without an opticospinal inflammatory event. These antibodies can be associated with other neurological syndromes such as intractable hiccups and vomiting due to lesions in the *area postrema*, highlighting the broadening spectrum of AQP4-associated neurological disease, both with and without lupus (45, 76). Spinal cord disease in SLE is heterogeneous and short transverse myelitis and ischemic transverse myelitis can also occur (22, 77). Our increased understanding of the pathogenesis of spinal cord disease in lupus highlights that a myelopathic presentation can be caused by multiple different etiologies (77), with diverse treatment options (23), requiring careful evaluation (Figure 2C).

#### Meningitis

Meningitis, as described in the ACR case definitions, specifically refers to an autoimmune aseptic meningitis. This can occur in lupus patients in isolation, but can also accompany other events such transverse myelitis (78). It is rare. Given that many individuals with lupus are immunosuppressed, a critical differential diagnosis is one of infectious meningitis caused by typical or opportunistic pathogens. A broad spectrum of pathogens including *Cryptococcus neoformans* and *Listeria monocytogenes* can cause meningitis in lupus patients and microbiological advice should be sought (78). The clinical presentation of opportunistic organisms may vary, for example, fungal meningitis or listeriosis may present with raised intracranial pressure and cranial neuropathies rather than meningism and fever (78). Aseptic meningitis has also been described as a consequence of drugs used to treat lupus, including NSAIDs (79).

#### Movement Disorders

Chorea, a hyperkinetic movement disorder, has been reported in lupus patients (80), although reversible forms of parkinsonism, a hypokinetic movement disorder, has also been described, particularly in young-onset disease (81, 82). Myoclonus has also been described (83). The etiology of these movement disorders is poorly understood and neuroimaging studies do not usually identify evidence of a localizing lesion (84). Both ischemic and antibody-mediated causes have been postulated, though not convincingly demonstrated.

#### Demyelinating Syndrome

An association between lupus and MS-like brain changes have been suggested, and sometimes termed “lupoid sclerosis” (85). However, many such studies pre-date high quality MRI brain imaging which has greatly facilitated accurate diagnosis of MS. Much of this confusion stems from the superficial similarities between the presence of small white matter lesions on the MRI brain scans of patients with both MS and lupus. Advances in our understanding of the pathogenesis of MS in the past decades highlight that these lesions are distinct from those observed in lupus (86). Lesions in MS can usually be distinguished from those of lupus with MRI brain imaging. For example, lesions in lupus rarely enhance and correlate at a pathological level with small vessel injury (54), rather than the lymphocytic infiltration and demyelination seen in MS lesions (42, 86). Active MS lesions often display incomplete ring enhancement, and typically occur in a more periventricular distribution. True co-existence of lupus and MS is uncommon (19, 87), and there is no convincing evidence that lupus can cause an MS-like syndrome (87). In patients with both lupus and convincing clinical and paraclinical evidence of MS (88), a more plausible explanation is that, as is sometimes seen autoimmunity, the two diseases co-exist in a single individual (89). This presents a specific management challenge of identifying immunotherapies that might offer efficacy against both diseases.

#### Headache

Headache is a highly prevalent disorder in people with SLE (90), but there is no convincing evidence that this incidence is higher than that seen in the general population (91). Thus the entity of “lupus headache” is controversial (92). Headache in individuals with lupus should be approached in the same way as in the general population, noting the broader differential diagnosis of any new acute headache to include a higher risk of infectious and neurovascular etiologies (64).

#### Psychiatric Disease

The term “lupus psychosis” has been used to describe single or repeated episodes of thought disorders such as hallucinations and delusions occurring in people with SLE (93, 94). Like many neuropsychiatric symptoms, the biology of psychosis remains poorly understood, although the possibility of an autoimmune contribution is the subject of intense current research interest (47, 95). Individuals with lupus are exposed to a number of biological substances which can cause psychosis, in particular corticosteroids and circulating antibodies directed against the NMDA-R (47). An association has also been identified between psychosis in lupus and anti-ribosomal-P antibodies (96), which can react against neuronal cell surface antigens (97). However, while antibodies directed against dsDNA, NMDA-R, and ribosomal-P may exhibit some neurotoxic effects in adoptive transfer experiments, their role in mediating psychiatric symptomatology and other brain symptoms in humans is not clear (98). A proportion of psychotic events in lupus are temporally related to corticosteroid use, although such observations are likely to be confounded by increases in systemic disease activity which might precede increased steroid dose (99–101). Differentiation of steroid-induced psychosis from lupus-associated psychosis is particularly challenging (100).

Depression and anxiety are common in the general population and observed more frequently in chronic disease states. It is, therefore, not surprising that about 15% of patients diagnosed with lupus develop mood disorders and 5% an anxiety disorder (12, 102). However, the use of both interviews and validated scales to quantify affective disorders suggest that the prevalence of mood and anxiety disorders may be significantly higher, around 20–40% (103–106). It has been established in clinical trials of therapeutic cytokines that inflammatory factors, such as type I interferon proteins, can induce depressive illness in humans (36, 107). Therefore, the degree to which lupus-related inflammatory factors contribute to the high burden of psychiatric disorders in this condition remains unresolved.

#### Cognitive Dysfunction

Longitudinal cognitive assessment in people with SLE show that cognition can vary over time (108, 109), though true dementia is not common (110). There is no clear association with lupus activity (111). Screening tools are of use to identify cognitive dysfunction in the clinic and should prompt more detailed neuropsychological testing if abnormal (112). However, cognitive changes can be transient and their substrate poorly defined. While some correlation with MRI abnormalities has been identified, this is not a robust association (113). Associations with elevated cytokines such as IL-6 have also been identified, but again a causal relationship is unclear (38). Evaluation of cognitive symptoms in people with lupus requires careful clinical evaluation, paying attention to additional factors such as depression and medication which can contribute to cognitive dysfunction. Neither corticosteroids (114) nor NMDA-R antagonists (115) have been shown to improve cognitive functioning in SLE, though cognitive rehabilitation approaches have shown some promise (116).

#### Rare Entities

Posterior reversible encephalopathy syndrome is a clinical–radiological syndrome of headache, seizures, and encephalopathy associated with white matter changes which occur mainly toward the posterior regions of the brain (117). Despite its name, the neurological damage caused by PRES is not necessarily reversible and can occur throughout the brain. A number of cases of PRES in people with SLE have been reported (71), but this syndrome can be confounded by associations with immunosuppressive medications and uncontrolled hypertension, and, therefore, the precise etiological factors are not fully understood (71). PRES-like appearances on neuroimaging can be mimicked by venous sinus thrombosis, which is an important differential diagnosis.

Another rare manifestation of lupus is the macrophage activation syndrome which can occur with prominent neurological involvement including seizures and encephalopathy (70). This is an important differential diagnosis of the acutely unwell lupus patient with multisystem involvement and requires prompt identification and treatment.

#### Inflammatory Neuromuscular Disease

Neuromuscular disease is an important cause of morbidity in SLE. The ACR neurolupus case definitions consider cranial nerve, peripheral nerve, and neuromuscular junction disease together, stopping at the motor end-plate and excluding muscle disease, which is classified separately. Muscle disease is, therefore, not reviewed in depth here, although it should be noted that a spectrum of inflammatory muscle disease can occur in about 10% of patients with SLE, including myositis and vasculitis, sometimes requiring biopsy confirmation (118–120).

#### Peripheral Neuropathy

Peripheral neuropathy can occur in approximately 8% of patients with lupus, presenting mainly as a symmetrical polyneuropathy (121, 122). Mononeuritis multiplex can also occur occasionally in lupus and is associated with small vessel vasculitic change on nerve biopsy, often developing during periods of high lupus activity (123, 124). Prospective studies, based on electrophysiological studies rather than symptoms, suggest that the commonest electrophysiological pattern is that of a sensorimotor axonal neuropathy (122). Among lupus-associated neuropathies, the identification of demyelinating inflammatory neuropathies is of particular importance, given the demonstrated response of such neuropathies to intravenous immunoglobulin (125). Identification of inflammatory demyelination on nerve conduction studies should provoke examination of the CSF and a search for paraproteinemic comorbidities (126). Very rarely, Guillain–Barré Syndrome—an acute inflammatory neuropathy—has been observed (127) as has myasthenia gravis.

#### Cranial Neuropathy

Optic neuropathies, manifesting as either optic neuritis or ischemic optic neuropathy, have been observed in SLE (128–132). Given the association of NMOSD with lupus, evaluation of anti-AQP4/MOG antibodies is important and may potentially guide treatment (74, 133). Cranial neuropathies affecting all cranial nerves have been reported in lupus (134–137), either as single events or as a cranial mononeuritis multiplex (137, 138).

#### Functional Disorders

Functional symptoms are real but are not caused by underlying neurological disease. Functional neurological disorder is a common cause of neurological symptoms, in both general medicine and neurology clinics, and can, therefore, frequently co-exist with inflammatory diseases such as lupus (139). Incorrectly attributing functional symptoms to an inflammatory cause can lead to an inappropriate escalation in immunotherapy or unnecessary investigation. A specialist neurological opinion can help to identify positive findings of functional neurological disease. The incidence of functional symptomatology in lupus and other inflammatory diseases is unknown and merits further study (139).

### Treatment of Neurolupus

While efforts have been made to guide best practice in the diagnosis and management of neurolupus, there is only a weak evidence base on which to develop such recommendations (55). There have been a handful of clinical studies for the treatment of lupus-associated neurological disease, none which provide high quality evidence. A small randomized trial of cyclophosphamide suggested potential benefit, but interpretation of these data are limited by small sample size and methodological issues (140, 141). There have also been observational studies of azathioprine (142) and rituximab (143), but the high degree of variability of clinical symptomatology and a lack of standardized neurological outcome measures makes these results difficult to interpret. Furthermore, meaningful metrics of neurological disease are rarely captured in large lupus clinical trials, and patients with neurological disease are often excluded from such studies (144).

## Future Directions

**Figure 4:**
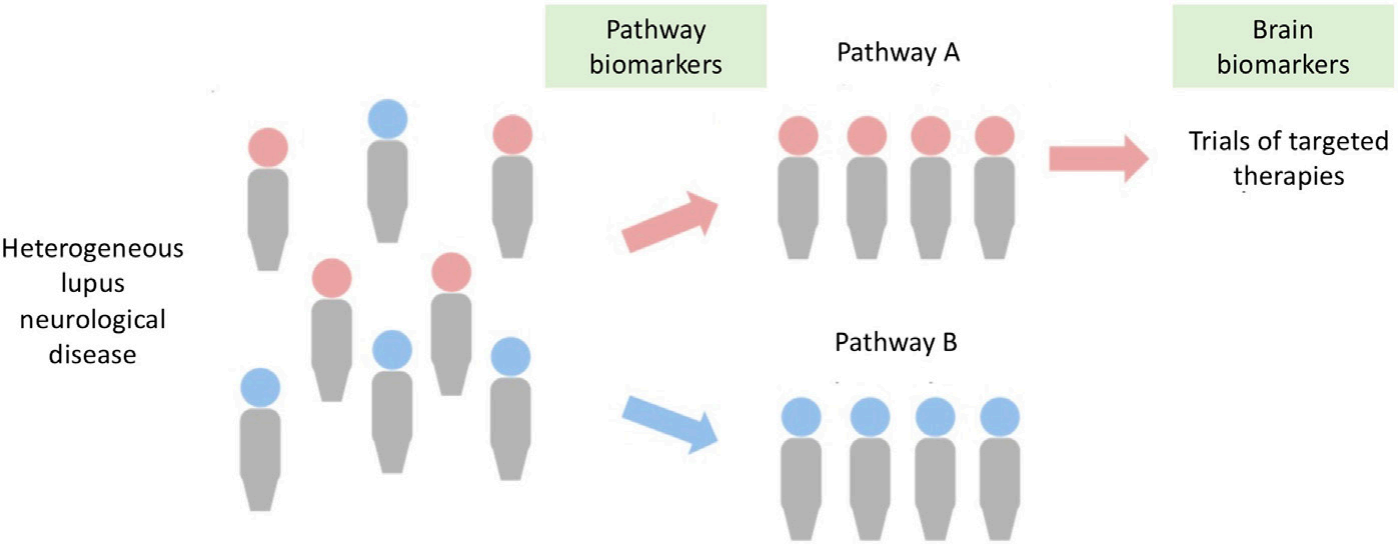
A stratified medicine approach for neurolupus. Brain disease in lupus is clinically heterogeneous (left), but may be driven by certain molecular pathways (e.g., type I interferon pathway, pathogenic autoantibodies), allowing stratification of populations. Improvements in biomarkers will allow identification of aberrant pathways in patients, such that they can be directed to clinical trials targeted at the specific pathway. Central to the success of such a strategy is the development of brain biomarkers (e.g., magnetic resonance imaging scans, markers of brain damage) to supplement clinical assessment.

Systemic lupus erythematosus is a strong candidate for a “personalized immunotherapy” approach, since individual patients may have different molecular pathways driving their disease. Longitudinal studies of lupus patients, together with their peripheral transcriptomic responses, support this approach to developing targeted therapies. These analyses show that targetable pathways—or combinations of pathways—can drive different aspects of lupus (33). For example, activation of the type I interferon response is an important determinant of organ-specific disease and is implicated in aspects of brain disease. Similarly, B-cell pathways play an important role in neurological syndromes caused by pathogenic autoantibodies. Thus, with the advent of more accurate biomarkers to identify aberrant immunological pathways, heterogeneous populations could be divided into those who are predicted to respond to targeted therapies, acting as a basis for rational trial design (Figure 4) (32, 37, 145). If this approach is to provide a logical framework for developing therapies, then we need to incorporate such a molecular understanding into clinical classification.

At present, the classification system for neurological disease in lupus is largely based on neurological syndromes and does not incorporate a pathophysiological understanding of the disease (Figure 2). The need to move from a syndromic toward a mechanistic classification is perhaps best exemplified by spinal cord disease in lupus (Figure 2C). The 1999 ACR case definitions refer to a broad syndrome of “lupus myelopathy.” However, as we describe above, our understanding of the pathogenesis of spinal cord disease in lupus has advanced, together with the discovery of strong biomarkers and improved imaging. It is clear that “lupus myelopathy” can be caused by at least three different pathophysiological processes. These include antibodies against AQP4, antibody-independent inflammation, and spinal vascular disease. It is likely that each of these different mechanisms may require a different therapeutic approach. Furthermore, some syndromes, such as “lupus headache,” may not exist at all. As such the classification system used in neurolupus requires substantial revision, reflecting the transition to a molecular understanding of disease.

A critical step in the future success of neurolupus clinical trials will be improving the quantification of neurological outcomes. There is a particular need to develop validated imaging and laboratory biomarkers of neurological disease in lupus which can supplement complex clinical assessment. MRI brain scans are invariably abnormal in lupus, and change over time. As such, imaging biomarkers may play a role as our ability to quantify macrostructural and microstructural damage (Figure 3). Serum and CSF biomarkers of “brain damage,” such as ultrasensitive detection of neurofilament protein, have been developed as a surrogate marker for clinical trials in neuroinflammatory and neurodegenerative diseases (146). Thus the rapid progress in our understanding of both pathophysiology and biomarkers of neurolupus is providing a much-needed roadmap to advance the field.

## Summary

Neurological disease is an area of major unmet need for people with lupus, providing a complex conceptual and practical challenge. An improved molecular understanding of how lupus can damage the brain and nervous system is providing opportunities to pursue stratified medicine approaches. Advancing the field will require our tools for classifying and measuring neurological disease in lupus to be reevaluated.

## Author Contributions

DH and SM drafted the original manuscript. Further revisions were made by SW, ND, and JW. SW and JW provided additional images.

## Conflict of Interest Statement

The authors declare that the research was conducted in the absence of any commercial or financial relationships that could be construed as a potential conflict of interest. The reviewer VK and handling Editor declared their shared affiliation.

## References

[1] Tsokos GC Systemic lupus erythematosus. N Engl J Med (2011) 365(22):2110–21. 10.1056/NEJMra1100359 22129255

[2] Kaposi M Lupus erythematosus. In: Hebra H Kaposi M, editors. Diseases of the Skin Including the Exanthemata. Vol IV. 1875 London: The New Sydenham Society (1880), p. 14–37. (transl. By Tay W).

[3] Osler W On the visceral complications of erythema exudativum multiforme. Am J Med Sci (1895) 110:629–46. 10.1097/00000441-189512000-00001 769549

[4] Daly D Central nervous system in acute disseminate lupus erythematosus. J Nerv Ment Dis (1945) 102:461–5. 10.1097/00005053-194511000-00005 21004281

[5] Kassan SS Lockshin MD Central nervous system lupus erythematosus. The need for classification. Arthritis Rheum (1979) 22(12):1382–5. 10.1002/art.1780221210 518720

[6] The American college of rheumatology nomenclature and case definitions for neuropsychiatric lupus syndromes. Arthritis Rheum (1999) 42(4):599–608. 10211873 10.1002/1529-0131(199904)42:4<599::AID-ANR2>3.0.CO;2-F

[7] Hanly JG. ACR classification criteria for systemic lupus erythematosus: limitations and revisions to neuropsychiatric variables. Lupus (2004) 13(11):861–4. 10.1191/0961203304lu2024oa 15580983

[8] Nived O Sturfelt G Liang MH De Pablo P. The ACR nomenclature for CNS lupus revisited. Lupus (2003) 12(12):872–6. 10.1191/0961203303lu495oa 14714904

[9] Petri M Orbai AM Alarcon GS Gordon C Merrill JT Fortin PR Derivation and validation of the systemic lupus international collaborating clinics classification criteria for systemic lupus erythematosus. Arthritis Rheum (2012) 64(8):2677–86. 10.1002/art.34473 22553077 PMC3409311

[10] Gladman D Ginzler E Goldsmith C Fortin P Liang M Urowitz M The development and initial validation of the systemic lupus international collaborating clinics/American college of rheumatology damage index for systemic lupus erythematosus. Arthritis Rheum (1996) 39(3):363–9. 10.1002/art.1780390303 8607884

[11] Brey RL Holliday SL Saklad AR Navarrete MG Hermosillo-Romo D Stallworth CL Neuropsychiatric syndromes in lupus: prevalence using standardized definitions. Neurology (2002) 58(8):1214–20. 10.1212/WNL.58.8.1214 11971089

[12] Hanly JG Urowitz MB Su L Bae SC Gordon C Wallace DJ Prospective analysis of neuropsychiatric events in an international disease inception cohort of patients with systemic lupus erythematosus. Ann Rheum Dis (2010) 69(3):529–35. 10.1136/ard.2008.106351 19359262 PMC2929162

[13] Hanly JG Urowitz MB Su L Sanchez-Guerrero J Bae SC Gordon C Short-term outcome of neuropsychiatric events in systemic lupus erythematosus upon enrollment into an international inception cohort study. Arthritis Rheum (2008) 59(5):721–9. 10.1002/art.23566 18438902 PMC4656032

[14] Bortoluzzi A Scire CA Bombardieri S Caniatti L Conti F De Vita S Development and validation of a new algorithm for attribution of neuropsychiatric events in systemic lupus erythematosus. Rheumatology (2015) 54(5):891–8. 10.1093/rheumatology/keu384 25339643

[15] Ainiala H Hietaharju A Loukkola J Peltola J Korpela M Metsanoja R Validity of the new American college of rheumatology criteria for neuropsychiatric lupus syndromes: a population-based evaluation. Arthritis Rheum (2001) 45(5):419–23. 10.1002/1529-0131(200110)45:5<419::AID-ART360>3.0.CO;2-X 11642640

[16] Hanly JG Urowitz MB Sanchez-Guerrero J Bae SC Gordon C Wallace DJ Neuropsychiatric events at the time of diagnosis of systemic lupus erythematosus: an international inception cohort study. Arthritis Rheum (2007) 56(1):265–73. 10.1002/art.22305 17195230

[17] Kampylafka EI Alexopoulos H Kosmidis ML Panagiotakos DB Vlachoyiannopoulos PG Dalakas MC Incidence and prevalence of major central nervous system involvement in systemic lupus erythematosus: a 3-year prospective study of 370 patients. PLoS One (2013) 8(2):e55843. 10.1371/journal.pone.0055843 23424638 PMC3570560

[18] Wiseman SJ Bastin ME Jardine CL Barclay G Hamilton IF Sandeman E Cerebral small vessel disease burden is increased in systemic lupus erythematosus. Stroke (2016) 47(11):2722–8. 10.1161/STROKEAHA.116.014330 27703087 PMC5079231

[19] Al-Obaidi M Saunders D Brown S Ramsden L Martin N Moraitis E Evaluation of magnetic resonance imaging abnormalities in juvenile onset neuropsychiatric systemic lupus erythematosus. Clin Rheumatol (2016) 35(10):2449–56. 10.1007/s10067-016-3376-9 27527090 PMC5031744

[20] Weening JJ D’Agati VD Schwartz MM Seshan SV Alpers CE Appel GB The classification of glomerulonephritis in systemic lupus erythematosus revisited. J Am Soc Nephrol (2004) 15(2):241–50. 10.1097/01.ASN.0000108969.21691.5D 14747370

[21] Asgari N Jarius S Laustrup H Skejoe HP Lillevang ST Weinshenker BG Aquaporin-4-autoimmunity in patients with systemic lupus erythematosus: a predominantly population-based study. Mult Scler (2017) 24(3):331–9. 10.1177/1352458517699791 28326889

[22] Provenzale J Bouldin TW. Lupus-related myelopathy: report of three cases and review of the literature. J Neurol Neurosurg Psychiatry (1992) 55(9):830–5. 10.1136/jnnp.55.9.830 1402976 PMC1015110

[23] Kovacs B Lafferty TL Brent LH DeHoratius RJ. Transverse myelopathy in systemic lupus erythematosus: an analysis of 14 cases and review of the literature. Ann Rheum Dis (2000) 59(2):120–4. 10.1136/ard.59.2.120 10666167 PMC1753077

[24] Bentham J Morris DL Graham DSC Pinder CL Tombleson P Behrens TW Genetic association analyses implicate aberrant regulation of innate and adaptive immunity genes in the pathogenesis of systemic lupus erythematosus. Nat Genet (2015) 47(12):1457–64. 10.1038/ng.3434 26502338 PMC4668589

[25] Lee-Kirsch MA Chowdhury D Harvey S Gong M Senenko L Engel K A mutation in TREX1 that impairs susceptibility to granzyme A-mediated cell death underlies familial chilblain lupus. J Mol Med (2007) 85(5):531–7. 10.1007/s00109-007-0199-9 17440703

[26] Lee-Kirsch MA Gong M Chowdhury D Senenko L Engel K Lee YA Mutations in the gene encoding the 3'-5' DNA exonuclease TREX1 are associated with systemic lupus erythematosus. Nat Genet (2007) 39(9):1065–7. 10.1038/ng2091 17660818

[27] Namjou B Kothari PH Kelly JA Glenn SB Ojwang JO Adler A Evaluation of the TREX1 gene in a large multi-ancestral lupus cohort. Genes Immun (2011) 12(4):270–9. 10.1038/gene.2010.73 21270825 PMC3107387

[28] Crow YJ Hayward BE Parmar R Robins P Leitch A Ali M Mutations in the gene encoding the 3'-5' DNA exonuclease TREX1 cause Aicardi-Goutieres syndrome at the AGS1 locus. Nat Genet (2006) 38(8):917–20. 10.1038/ng1845 16845398

[29] de Vries B Steup-Beekman GM Haan J Bollen EL Luyendijk J Frants RR TREX1 gene variant in neuropsychiatric systemic lupus erythematosus. Ann Rheum Dis (2010) 69(10):1886–7. 10.1136/ard.2009.114157 19875384

[30] McGlasson S Rannikmae K Bevan S Markus H Sudlow C Hunt DPJ Rare variants of the 3'-5' DNA exonuclease TREX1 in early onset small vessel stroke. Wellcome Open Res (2017) 2:106 10.12688/wellcomeopenres.12631.1 29387804 PMC5717473

[31] Lourenco EV La Cava A. Cytokines in systemic lupus erythematosus. Curr Mol Med (2009) 9(3):242–54. 10.2174/156652409787847263 19355907 PMC3589140

[32] Rodero MP Decalf J Bondet V Hunt D Rice GI Werneke S Detection of interferon alpha protein reveals differential levels and cellular sources in disease. J Exp Med (2017) 214(5):1547–55. 10.1084/jem.20161451 28420733 PMC5413335

[33] Banchereau R Hong S Cantarel B Baldwin N Baisch J Edens M Personalized immunomonitoring uncovers molecular networks that stratify lupus patients. Cell (2016) 165(3):551–65. 10.1016/j.cell.2016.03.008 27040498 PMC5426482

[34] Hunt D Kavanagh D Drummond I Weller B Bellamy C Overell J Thrombotic microangiopathy associated with interferon beta. N Engl J Med (2014) 370(13):1270–1. 10.1056/NEJMc1316118 24670186 PMC4066182

[35] Kavanagh D McGlasson S Jury A Williams J Scolding N Bellamy C Type I interferon causes thrombotic microangiopathy by a dose-dependent toxic effect on the microvasculature. Blood (2016) 128(24):2824–33. 10.1182/blood-2016-05-715987 27663672 PMC5159705

[36] Heinze S Egberts F Rotzer S Volkenandt M Tilgen W Linse R Depressive mood changes and psychiatric symptoms during 12-month low-dose interferon-alpha treatment in patients with malignant melanoma: results from the multicenter DeCOG trial. J Immunother (2010) 33(1):106–14. 10.1097/CJI.0b013e3181b8bdb9 19952950

[37] Bialas AR Presumey J Das A van der Poel CE Lapchak PH Mesin L Microglia-dependent synapse loss in type I interferon-mediated lupus. Nature (2017) 546(7659):539–43. 10.1038/nature22821 28614301

[38] Wiseman SJ Bastin ME Hamilton IF Hunt D Ritchie SJ Amft EN Fatigue and cognitive function in systemic lupus erythematosus: associations with white matter microstructural damage. A diffusion tensor MRI study and meta-analysis. Lupus (2017) 26(6):588–97. 10.1177/0961203316668417 27687026 PMC5374047

[39] Campbell IL Erta M Lim SL Frausto R May U Rose-John S Trans-signaling is a dominant mechanism for the pathogenic actions of interleukin-6 in the brain. J Neurosci (2014) 34(7):2503–13. 10.1523/JNEUROSCI.2830-13.2014 24523541 PMC6802757

[40] Jeltsch-David H Muller S Neuropsychiatric systemic lupus erythematosus: pathogenesis and biomarkers. Nat Rev Neurol (2014) 10(10):579–96. 10.1038/nrneurol.2014.148 25201240

[41] Scolding NJ Joseph FG. The neuropathology and pathogenesis of systemic lupus erythematosus. Neuropathol Appl Neurobiol (2002) 28(3):173–89. 10.1046/j.1365-2990.2002.00406.x 12060342

[42] Reich DS Lucchinetti CF Calabresi PA Multiple sclerosis. N Engl J Med (2018) 378(2):169–80. 10.1056/NEJMra1401483 29320652 PMC6942519

[43] Wang J Yang C Zhao Q Zhu Z Li Y Yang P. Microglia activation induced by serum of SLE patients. J Neuroimmunol (2017) 310:135–42. 10.1016/j.jneuroim.2017.07.010 28778438

[44] Hanly JG Fisk JD Eastwood B. Brain reactive autoantibodies and cognitive impairment in systemic lupus erythematosus. Lupus (1994) 3(3):193–9. 10.1177/096120339400300311 7951305

[45] Alexopoulos H Kampylafka EI Fouka P Tatouli I Akrivou S Politis PK Anti-aquaporin-4 autoantibodies in systemic lupus erythematosus persist for years and induce astrocytic cytotoxicity but not CNS disease. J Neuroimmunol (2015) 289:8–11. 10.1016/j.jneuroim.2015.10.007 26616866

[46] Dalmau J Gleichman AJ Hughes EG Rossi JE Peng X Lai M Anti-NMDA-receptor encephalitis: case series and analysis of the effects of antibodies. Lancet Neurol (2008) 7(12):1091–8. 10.1016/S1474-4422(08)70224-2 18851928 PMC2607118

[47] Lennox BR Pollak T Palmer-Cooper EC Scoriels L Harrison PJ Jones PB Serum neuronal cell-surface antibodies in first-episode psychosis-authors’ reply. Lancet Psychiatry (2017) 4(3):187–8. 10.1016/S2215-0366(17)30053-6 28236947

[48] DeGiorgio LA Konstantinov KN Lee SC Hardin JA Volpe BT Diamond B. A subset of lupus anti-DNA antibodies cross-reacts with the NR2 glutamate receptor in systemic lupus erythematosus. Nat Med (2001) 7(11):1189–93. 10.1038/nm1101-1189 11689882

[49] Ruiz-Irastorza G Crowther M Branch W Khamashta MA. Antiphospholipid syndrome. Lancet (2010) 376(9751):1498–509. 10.1016/S0140-6736(10)60709-X 20822807

[50] Johnson RT Richardson EP The neurological manifestations of systemic lupus erythematosus. Medicine (1968) 47(4):337–69. 10.1097/00005792-196807000-00002 5212395

[51] Joseph FG Scolding NJ. Neurolupus. Pract Neurol (2010) 10(1):4–15. 10.1136/jnnp.2009.200071 20130291

[52] Ellis SG Verity MA Central nervous system involvement in systemic lupus erythematosus: a review of neuropathologic findings in 57 cases, 1955–1977. Semin Arthritis Rheum (1979) 8(3):212–21. 10.1016/S0049-0172(79)80009-8 424765

[53] Hanly JG Walsh NM Sangalang V. Brain pathology in systemic lupus erythematosus. J Rheumatol (1992) 19(5):732–41. 1613703

[54] Sibbitt WL Jr Brooks WM Kornfeld M Hart BL Bankhurst AD Roldan CA. Magnetic resonance imaging and brain histopathology in neuropsychiatric systemic lupus erythematosus. Semin Arthritis Rheum (2010) 40(1):32–52. 10.1016/j.semarthrit.2009.08.005 19880162 PMC3586567

[55] Bertsias GK Ioannidis JP Aringer M Bollen E Bombardieri S Bruce IN EULAR recommendations for the management of systemic lupus erythematosus with neuropsychiatric manifestations: report of a task force of the EULAR standing committee for clinical affairs. Ann Rheum Dis (2010) 69(12):2074–82. 10.1136/ard.2010.130476 20724309

[56] Tay SH Mak A. Diagnosing and attributing neuropsychiatric events to systemic lupus erythematosus: time to untie the Gordian knot? Rheumatology (2017) 56(Suppl_1):i14–23. 10.1093/rheumatology/kex018 27744358

[57] Urowitz MB Gladman D Ibanez D Bae SC Sanchez-Guerrero J Gordon C Atherosclerotic vascular events in a multinational inception cohort of systemic lupus erythematosus. Arthritis Care Res (2010) 62(6):881–7. 10.1002/acr.20122 20535799 PMC2989413

[58] Mikdashi J Handwerger B Langenberg P Miller M Kittner S. Baseline disease activity, hyperlipidemia, and hypertension are predictive factors for ischemic stroke and stroke severity in systemic lupus erythematosus. Stroke (2007) 38(2):281–5. 10.1161/01.STR.0000254476.05620.14 17218611

[59] Chiu CC Huang CC Chan WL Chung CM Huang PH Lin SJ Increased risk of ischemic stroke in patients with systemic lupus erythematosus: a nationwide population-based study. Intern Med (2012) 51(1):17–21. 10.2169/internalmedicine.51.6154 22214618

[60] Bessant R Hingorani A Patel L MacGregor A Isenberg DA Rahman A. Risk of coronary heart disease and stroke in a large British cohort of patients with systemic lupus erythematosus. Rheumatology (2004) 43(7):924–9. 10.1093/rheumatology/keh213 15150430

[61] Wiseman SJ Ralston SH Wardlaw JM. Cerebrovascular disease in rheumatic diseases: a systematic review and meta-analysis. Stroke (2016) 47(4):943–50. 10.1161/STROKEAHA.115.012052 26917565

[62] Wiseman S Marlborough F Doubal F Webb DJ Wardlaw J. Blood markers of coagulation, fibrinolysis, endothelial dysfunction and inflammation in lacunar stroke versus non-lacunar stroke and non-stroke: systematic review and meta-analysis. Cerebrovasc Dis (2014) 37(1):64–75. 10.1159/000356789 24401164

[63] Harriott A Faye EC Abreu N Silverman S Rordorf G Aneurysmal subarachnoid and spinal hemorrhage associated with systemic lupus erythematosus. Stroke (2016) 47(3):e42–5. 26797667 10.1161/STROKEAHA.115.012373

[64] Mimori A Suzuki T Hashimoto M Nara H Yoshio T Masuyama JI Subarachnoid hemorrhage and systemic lupus erythematosus. Lupus (2000) 9(7):521–6. 10.1177/096120330000900708 11035418

[65] Kelley RE Stokes N Reyes P Harik SI. Cerebral transmural angiitis and ruptured aneurysm: a complication of systemic lupus erythematosus. Arch Neurol (1980) 37(8):526–7. 10.1001/archneur.1980.00500570074015 7417048

[66] Aribisala BS Wiseman S Morris Z Valdes-Hernandez MC Royle NA Maniega SM Circulating inflammatory markers are associated with magnetic resonance imaging-visible perivascular spaces but not directly with white matter hyperintensities. Stroke (2014) 45(2):605–7. 10.1161/STROKEAHA.113.004059 24399375 PMC3906539

[67] Bailey EL Smith C Sudlow CL Wardlaw JM Pathology of lacunar ischemic stroke in humans – a systematic review. Brain Pathol (2012) 22(5):583–91. 10.1111/j.1750-3639.2012.00575.x 22329603 PMC8057646

[68] Hanly JG Urowitz MB Su L Gordon C Bae SC Sanchez-Guerrero J Seizure disorders in systemic lupus erythematosus results from an international, prospective, inception cohort study. Ann Rheum Dis (2012) 71(9):1502–9. 10.1136/annrheumdis-2011-201089 22492779 PMC4656036

[69] Appenzeller S Cendes F Costallat LT. Epileptic seizures in systemic lupus erythematosus. Neurology (2004) 63(10):1808–12. 10.1212/01.WNL.0000144178.32208.4F 15557494

[70] Gavand PE Serio I Arnaud L Costedoat-Chalumeau N Carvelli J Dossier A Clinical spectrum and therapeutic management of systemic lupus erythematosus-associated macrophage activation syndrome: a study of 103 episodes in 89 adult patients. Autoimmun Rev (2017) 16(7):743–9. 10.1016/j.autrev.2017.05.010 28483541

[71] Shaharir SS Remli R Marwan AA Said MS Kong NC. Posterior reversible encephalopathy syndrome in systemic lupus erythematosus: pooled analysis of the literature reviews and report of six new cases. Lupus (2013) 22(5):492–6. 10.1177/0961203313478303 23435619

[72] Watad A Tiosano S Bragazzi NL Brigo F Comaneshter D Cohen AD Epilepsy among systemic lupus erythematosus patients: insights from a large database analysis. Neuroepidemiology (2017) 50(1–2):1–6. 10.1159/000485136 29208845

[73] Pittock SJ Lennon VA de Seze J Vermersch P Homburger HA Wingerchuk DM Neuromyelitis optica and non organ-specific autoimmunity. Arch Neurol (2008) 65(1):78–83. 10.1001/archneurol.2007.17 18195142

[74] Sellner J Boggild M Clanet M Hintzen RQ Illes Z Montalban X EFNS guidelines on diagnosis and management of neuromyelitis optica. Eur J Neurol (2010) 17(8):1019–32. 10.1111/j.1468-1331.2010.03066.x 20528913

[75] Damato V Evoli A Iorio R. Efficacy and safety of rituximab therapy in neuromyelitis optica spectrum disorders: a systematic review and meta-analysis. JAMA Neurol (2016) 73(11):1342–8. 10.1001/jamaneurol.2016.1637 27668357

[76] McKeon A Lennon VA Lotze T Tenenbaum S Ness JM Rensel M CNS aquaporin-4 autoimmunity in children. Neurology (2008) 71(2):93–100. 10.1212/01.wnl.0000314832.24682.c6 18509092

[77] Birnbaum J Petri M Thompson R Izbudak I Kerr D. Distinct subtypes of myelitis in systemic lupus erythematosus. Arthritis Rheum (2009) 60(11):3378–87. 10.1002/art.24937 19877037

[78] Baizabal-Carvallo JF Delgadillo-Marquez G Estanol B Garcia-Ramos G. Clinical characteristics and outcomes of the meningitides in systemic lupus erythematosus. Eur Neurol (2009) 61(3):143–8. 10.1159/000186504 19092250

[79] Faurie P Perard L Hot A Desmurs-Clavel H Fassier T Boibieux A Recurrent aseptic meningitis secondary to nonsteroidal anti-inflammatory drugs in a patient with lupus. Rev Med Interne (2010) 31(10):e1–3. 10.1016/j.revmed.2009.08.021 20541295

[80] Reiner P Galanaud D Leroux G Vidailhet M Haroche J Huong du LT Long-term outcome of 32 patients with chorea and systemic lupus erythematosus or antiphospholipid antibodies. Mov Disord (2011) 26(13):2422–7. 10.1002/mds.23863 21755538

[81] Khubchandani RP Viswanathan V Desai J. Unusual neurologic manifestations (I): parkinsonism in juvenile SLE. Lupus (2007) 16(8):572–5. 10.1177/0961203307081421 17711890

[82] Garcia-Moreno JM Chacon J. Juvenile parkinsonism as a manifestation of systemic lupus erythematosus: case report and review of the literature. Mov Disord (2002) 17(6):1329–35. 10.1002/mds.10288 12465077

[83] Joseph FG Lammie GA Scolding NJ. CNS lupus: a study of 41 patients. Neurology (2007) 69(7):644–54. 10.1212/01.wnl.0000267320.48939.d0 17698785

[84] Baizabal-Carvallo JF Bonnet C Jankovic J Movement disorders in systemic lupus erythematosus and the antiphospholipid syndrome. J Neural Transm (2013) 120(11):1579–89. 10.1007/s00702-013-1023-z 23580159

[85] Keiserman B da Silva LF Keiserman MW von Muhlen CA Staub HL. Lupoid sclerosis. Rheumatol Int (2010) 30(4):431–4. 10.1007/s00296-009-1175-1 19826821

[86] Frohman EM Racke MK Raine CS Multiple sclerosis – the plaque and its pathogenesis. N Engl J Med (2006) 354(9):942–55. 10.1056/NEJMra052130 16510748

[87] Fanouriakis A Mastorodemos V Pamfil C Papadaki E Sidiropoulos P Plaitakis A Coexistence of systemic lupus erythematosus and multiple sclerosis: prevalence, clinical characteristics, and natural history. Semin Arthritis Rheum (2014) 43(6):751–8. 10.1016/j.semarthrit.2013.11.007 24332007

[88] Thompson AJ Banwell BL Barkhof F Carroll WM Coetzee T Comi G Diagnosis of multiple sclerosis: 2017 revisions of the McDonald criteria. Lancet Neurol (2018) 17(2):162–73. 10.1016/S1474-4422(17)30470-2 29275977

[89] Jacome Sanchez EC Garcia Castillo MA Gonzalez VP Guillen Lopez F Correa Diaz EP. Coexistence of systemic lupus erythematosus and multiple sclerosis. A case report and literature review. Mult Scler J Exp Transl Clin (2018) 4(2):2055217318768330. 10.1177/2055217318768330 29662683 PMC5894926

[90] Hanly JG Urowitz MB O’Keeffe AG Gordon C Bae SC Sanchez-Guerrero J Headache in systemic lupus erythematosus: results from a prospective, international inception cohort study. Arthritis Rheum (2013) 65(11):2887–97. 10.1002/art.38106 24166793

[91] Davey R Bamford J Emery P. The ACR classification criteria for headache disorders in SLE fail to classify certain prevalent headache types. Cephalalgia (2008) 28(3):296–9. 10.1111/j.1468-2982.2007.01510.x 18254898

[92] Mitsikostas DD Katsiari C Sfikakis PP Lupus headache may not exist: comment on the article by Hanly et Al. Arthritis Rheumatol (2014) 66(4):1058 10.1002/art.38333 24757160

[93] Appenzeller S Cendes F Costallat LT. Acute psychosis in systemic lupus erythematosus. Rheumatol Int (2008) 28(3):237–43. 10.1007/s00296-007-0410-x 17634902

[94] Pego-Reigosa JM Isenberg DA. Psychosis due to systemic lupus erythematosus: characteristics and long-term outcome of this rare manifestation of the disease. Rheumatology (2008) 47(10):1498–502. 10.1093/rheumatology/ken260 18658205

[95] Al-Diwani AAJ Pollak TA Irani SR Lennox BR. Psychosis: an autoimmune disease? Immunology (2017) 152(3):388–401. 10.1111/imm.12795 28704576 PMC5629440

[96] Bonfa E Golombek SJ Kaufman LD Skelly S Weissbach H Brot N Association between lupus psychosis and anti-ribosomal P protein antibodies. N Engl J Med (1987) 317(5):265–71. 10.1056/NEJM198707303170503 3496538

[97] Matus S Burgos PV Bravo-Zehnder M Kraft R Porras OH Farias P Antiribosomal-P autoantibodies from psychiatric lupus target a novel neuronal surface protein causing calcium influx and apoptosis. J Exp Med (2007) 204(13):3221–34. 10.1084/jem.20071285 18056288 PMC2150977

[98] Karassa FB Afeltra A Ambrozic A Chang DM De Keyser F Doria A Accuracy of anti-ribosomal P protein antibody testing for the diagnosis of neuropsychiatric systemic lupus erythematosus: an international meta-analysis. Arthritis Rheum (2006) 54(1):312–24. 10.1002/art.21539 16385548

[99] Nishimura K Omori M Sato E Katsumata Y Gono T Kawaguchi Y New-onset psychiatric disorders after corticosteroid therapy in systemic lupus erythematosus: an observational case-series study. J Neurol (2014) 261(11):2150–8. 10.1007/s00415-014-7472-y 25142268

[100] Bhangle SD Kramer N Rosenstein ED. Corticosteroid-induced neuropsychiatric disorders: review and contrast with neuropsychiatric lupus. Rheumatol Int (2013) 33(8):1923–32. 10.1007/s00296-013-2750-z 23588411

[101] Shimizu Y Yasuda S Kako Y Nakagawa S Kanda M Hisada R Post-steroid neuropsychiatric manifestations are significantly more frequent in SLE compared with other systemic autoimmune diseases and predict better prognosis compared with de novo neuropsychiatric SLE. Autoimmun Rev (2016) 15(8):786–94. 10.1016/j.autrev.2016.03.017 27016478

[102] Hanly JG Su L Urowitz MB Romero-Diaz J Gordon C Bae SC Mood disorders in systemic lupus erythematosus: results from an international inception cohort study. Arthritis Rheumatol (2015) 67(7):1837–47. 10.1002/art.39111 25778456 PMC4485527

[103] Iverson GL Sawyer DC McCracken LM Kozora E. Assessing depression in systemic lupus erythematosus: determining reliable change. Lupus (2001) 10(4):266–71. 10.1191/096120301680416959 11341103

[104] Kozora E Ellison MC West S. Depression, fatigue, and pain in systemic lupus erythematosus (SLE): relationship to the American college of rheumatology SLE neuropsychological battery. Arthritis Rheum (2006) 55(4):628–35. 10.1002/art.22101 16874786

[105] Kozora E Arciniegas DB Zhang L West S Neuropsychological patterns in systemic lupus erythematosus patients with depression. Arthritis Res Ther (2007) 9(3):R48 10.1186/ar2203 17504538 PMC2206349

[106] Zhang L Fu T Yin R Zhang Q Shen B. Prevalence of depression and anxiety in systemic lupus erythematosus: a systematic review and meta-analysis. BMC Psychiatry (2017) 17(1):70. 10.1186/s12888-017-1234-1 28196529 PMC5310017

[107] Leighton SP Nerurkar L Krishnadas R Johnman C Graham GJ Cavanagh J. Chemokines in depression in health and in inflammatory illness: a systematic review and meta-analysis. Mol Psychiatry (2018) 23(1):48–58. 10.1038/mp.2017.205 29133955 PMC5754468

[108] Hanly JG Cassell K Fisk JD Cognitive function in systemic lupus erythematosus: results of a 5-year prospective study. Arthritis Rheum (1997) 40(8):1542–3. 10.1002/art.1780400825 9259438

[109] Waterloo K Omdal R Husby G Mellgren SI. Neuropsychological function in systemic lupus erythematosus: a five-year longitudinal study. Rheumatology (2002) 41(4):411–5. 10.1093/rheumatology/41.4.411 11961171

[110] Hanly JG Fisk JD Diagnosis of cognitive impairment in adult and pediatric SLE. Nat Rev Rheumatol (2011) 7(10):564–5. 10.1038/nrrheum.2011.127 21862982

[111] Glanz BI Slonim D Urowitz MB Gladman DD Gough J MacKinnon A. Pattern of neuropsychologic dysfunction in inactive systemic lupus erythematosus. Neuropsychiatry Neuropsychol Behav Neurol (1997) 10(4):232–8. 9359119

[112] Kozora E Ellison MC West S. Reliability and validity of the proposed American college of rheumatology neuropsychological battery for systemic lupus erythematosus. Arthritis Rheum (2004) 51(5):810–8. 10.1002/art.20692 15478145

[113] Ainiala H Dastidar P Loukkola J Lehtimaki T Korpela M Peltola J Cerebral MRI abnormalities and their association with neuropsychiatric manifestations in SLE: a population-based study. Scand J Rheumatol (2005) 34(5):376–82. 10.1080/03009740510026643 16234185

[114] Denburg SD Carbotte RM Denburg JA. Corticosteroids and neuropsychological functioning in patients with systemic lupus erythematosus. Arthritis Rheum (1994) 37(9):1311–20. 10.1002/art.1780370907 7945494

[115] Petri M Naqibuddin M Sampedro M Omdal R Carson KA. Memantine in systemic lupus erythematosus: a randomized, double-blind placebo-controlled trial. Semin Arthritis Rheum (2011) 41(2):194–202. 10.1016/j.semarthrit.2011.02.005 21458845 PMC3153605

[116] Harrison MJ Morris KA Horton R Toglia J Barsky J Chait S Results of intervention for lupus patients with self-perceived cognitive difficulties. Neurology (2005) 65(8):1325–7. 10.1212/01.wnl.0000180938.69146.5e 16247073

[117] Baizabal-Carvallo JF Barragan-Campos HM Padilla-Aranda HJ Alonso-Juarez M Estanol B Cantu-Brito C Posterior reversible encephalopathy syndrome as a complication of acute lupus activity. Clin Neurol Neurosurg (2009) 111(4):359–63. 10.1016/j.clineuro.2008.11.017 19128872

[118] Isenber DA Snaith ML. Muscle Disease in systemic lupus erythematosus: a study of its nature, frequency and cause. J Rheumatol (1981) 8(6):917–24. 7328567

[119] Lim KL Abdul-Wahab R Lowe J Powell RJ. Muscle biopsy abnormalities in systemic lupus erythematosus: correlation with clinical and laboratory parameters. Ann Rheum Dis (1994) 53(3):178–82. 10.1136/ard.53.3.178 8154935 PMC1005282

[120] Tsokos GC Moutsopoulos HM Steinberg AD Muscle involvement in systemic lupus erythematosus. JAMA (1981) 246(7):766–8. 10.1001/jama.1981.03320070050025 7253141

[121] Florica B Aghdassi E Su J Gladman DD Urowitz MB Fortin PR. Peripheral neuropathy in patients with systemic lupus erythematosus. Semin Arthritis Rheum (2011) 41(2):203–11. 10.1016/j.semarthrit.2011.04.001 21641018

[122] Omdal R Loseth S Torbergsen T Koldingsnes W Husby G Mellgren SI Peripheral neuropathy in systemic lupus erythematosus – a longitudinal study. Acta Neurol Scand (2001) 103(6):386–91. 10.1034/j.1600-0404.2001.103006386.x 11421851

[123] Hellmann DB Laing TJ Petri M Whiting-O’Keefe Q Parry GJ. Mononeuritis multiplex: the yield of evaluations for occult rheumatic diseases. Medicine (1988) 67(3):145–53. 10.1097/00005792-198805000-00001 2835572

[124] Riviere E Cohen Aubart F Maisonobe T Maurier F Richez C Gombert B Clinicopathological features of multiple mononeuropathy associated with systemic lupus erythematosus: a multicenter study. J Neurol (2017) 264(6):1218–26. 10.1007/s00415-017-8519-7 28536920

[125] Hughes RA Donofrio P Bril V Dalakas MC Deng C Hanna K Intravenous immune globulin (10% caprylate-chromatography purified) for the treatment of chronic inflammatory demyelinating polyradiculoneuropathy (ICE study): a randomised placebo-controlled trial. Lancet Neurol (2008) 7(2):136–44. 10.1016/S1474-4422(07)70329-0 18178525

[126] Vina ER Fang AJ Wallace DJ Weisman MH. Chronic inflammatory demyelinating polyneuropathy in patients with systemic lupus erythematosus: prognosis and outcome. Semin Arthritis Rheum (2005) 35(3):175–84. 10.1016/j.semarthrit.2005.08.008 16325658

[127] Toledano P Orueta R Rodriguez-Pinto I Valls-Sole J Cervera R Espinosa G. Peripheral nervous system involvement in systemic lupus erythematosus: prevalence, clinical and immunological characteristics, treatment and outcome of a large cohort from a single centre. Autoimmun Rev (2017) 16(7):750–5. 10.1016/j.autrev.2017.05.011 28483540

[128] Frigui M Frikha F Sellemi D Chouayakh F Feki J Bahloul Z. Optic neuropathy as a presenting feature of systemic lupus erythematosus: two case reports and literature review. Lupus (2011) 20(11):1214–8. 10.1177/0961203311403344 21669911

[129] Teoh SC Yap EY Au Eong KG. Neuro-ophthalmological manifestations of systemic lupus erythematosus in Asian patients. Clin Exp Ophthalmol (2001) 29(4):213–6. 10.1046/j.1442-9071.2001.00424.x 11545417

[130] Lin YC Wang AG Yen MY. Systemic lupus erythematosus-associated optic neuritis: clinical experience and literature review. Acta Ophthalmol (2009) 87(2):204–10. 10.1111/j.1755-3768.2008.01193.x 18507726

[131] Giorgi D Balacco Gabrieli C. Optic neuropathy in systemic lupus erythematosus and antiphospholipid syndrome (APS): clinical features, pathogenesis, review of the literature and proposed ophthalmological criteria for APS diagnosis. Clin Rheumatol (1999) 18(2):124–31. 10.1007/s100670050069 10357117

[132] Giorgi D Balacco Gabrieli C Bonomo L The association of optic neuropathy with transverse myelitis in systemic lupus erythematosus. Rheumatology (1999) 38(2):191–2. 10.1093/rheumatology/38.2.191 10342640

[133] Trebst C Jarius S Berthele A Paul F Schippling S Wildemann B Update on the diagnosis and treatment of neuromyelitis optica: recommendations of the neuromyelitis optica study group (NEMOS). J Neurol (2014) 261(1):1–16. 10.1007/s00415-013-7169-7 24272588 PMC3895189

[134] Genevay S Hayem G Hamza S Palazzo E Meyer O Kahn MF. Oculomotor palsy in six patients with systemic lupus erythematosus. A possible role of antiphospholipid syndrome. Lupus (2002) 11(5):313–6. 10.1191/0961203302lu205oa 12090567

[135] Hughes M Hill J. Left vocal cord paralysis in systemic lupus erythematosus. Mod Rheumatol (2009) 19(4):441–2. 10.1007/s10165-009-0178-9 19458907

[136] Lorenzoni PJ Scola RH Kay CS Novak FT Cardoso EH Scalcon MR Isolated hypoglossal nerve palsy: an unusual rare presentation in systemic lupus erythematosus. Arq Neuropsiquiatr (2011) 69(5):843–4. 10.1590/S0004-282X2011000600025 22042195

[137] Gaber W Ezzat Y El Fayoumy NM Helmy H Mohey AM. Detection of asymptomatic cranial neuropathies in patients with systemic lupus erythematosus and their relation to antiribosomal P antibody levels and disease activity. Clin Rheumatol (2014) 33(10):1459–66. 10.1007/s10067-014-2679-y 24852670

[138] Crespo Cuevas AM Hervas Garcia JV Abraira Del Fresno L Grau Lopez L Cranial mononeuritis multiplex as the initial manifestation of systemic lupus erythematosus: a diagnostic challenge. Neurologia (2016) 33(2):135–7. 10.1016/j.nrl.2016.01.003 26971057

[139] Stone J The bare essentials: functional symptoms in neurology. Pract Neurol (2009) 9(3):179–89. 10.1136/jnnp.2009.177204 19448064

[140] Barile-Fabris L Ariza-Andraca R Olguin-Ortega L Jara LJ Fraga-Mouret A Miranda-Limon JM Controlled clinical trial of IV cyclophosphamide versus IV methylprednisolone in severe neurological manifestations in systemic lupus erythematosus. Ann Rheum Dis (2005) 64(4):620–5. 10.1136/ard.2004.025528 15769918 PMC1755456

[141] Trevisani VF Castro AA Neves Neto JF Atallah AN Cyclophosphamide versus methylprednisolone for treating neuropsychiatric involvement in systemic lupus erythematosus. Cochrane Database Syst Rev (2006) 2:CD002265. 10.1002/14651858.CD002265.pub2 16625558

[142] Mok CC Lau CS Wong RW Treatment of lupus psychosis with oral cyclophosphamide followed by azathioprine maintenance: an open-label study. Am J Med (2003) 115(1):59–62. 10.1016/S0002-9343(03)00135-9 12867236

[143] Tokunaga M Saito K Kawabata D Imura Y Fujii T Nakayamada S Efficacy of rituximab (anti-CD20) for refractory systemic lupus erythematosus involving the central nervous system. Ann Rheum Dis (2007) 66(4):470–5. 10.1136/ard.2006.057885 17107983 PMC1856059

[144] Navarra SV Guzman RM Gallacher AE Hall S Levy RA Jimenez RE Efficacy and safety of belimumab in patients with active systemic lupus erythematosus: a randomised, placebo-controlled, phase 3 trial. Lancet (2011) 377(9767):721–31. 10.1016/S0140-6736(10)61354-2 21296403

[145] McGlasson S Hunt D Neuroinflammation: synapses pruned in lupus. Nature (2017) 546(7659):482–3. 28614295 10.1038/nature23087

[146] Johnson EB Byrne LM Gregory S Rodrigues FB Blennow K Durr A Neurofilament light protein in blood predicts regional atrophy in Huntington disease. Neurology (2018). 10.1212/WNL.0000000000005005 29367444 PMC5818166

[147] Guerra H Pittock SJ Moder KG Fryer JP Gadoth A Flanagan EP. Frequency of aquaporin-4 immunoglobulin G in longitudinally extensive transverse myelitis with antiphospholipid antibodies. Mayo Clin Proc (2018). 10.1016/j.mayocp.2018.02.006 29655487

[148] Mader S Jeganathan V Arinuma Y Fujieda Y Dujmovic I Drulovic J Understanding the antibody repertoire in neuropsychiatric systemic lupus erythematosus and neuromyelitis optica spectrum disorder: do they share common targets? Arthritis Rheumatol (2018) 70(2):277–86. 10.1002/art.40356 29073350

[149] Alpa M Ferrero B Cavallo R Perna A Naretto C Gennaro M Anti-GM1 and anti-sulfatide antibodies in patients with systemic lupus erythematosus, Sjogren’s syndrome, mixed cryoglobulinemia and idiopathic systemic vasculitis. Clin Exp Rheumatol (2007) 25(4):556–62. 17888211

[150] Kovacs KT Kalluri SR Boza-Serrano A Deierborg T Csepany T Simo M Change in autoantibody and cytokine responses during the evolution of neuromyelitis optica in patients with systemic lupus erythematosus: a preliminary study. Mult Scler (2016) 22(9):1192–201. 10.1177/1352458515613165 26514978

[151] Nacu A Andersen JB Lisnic V Owe JF Gilhus NE. Complicating autoimmune diseases in myasthenia gravis: a review. Autoimmunity (2015) 48(6):362–8. 10.3109/08916934.2015.1030614 25915571 PMC4616023

